# Hemoglobin Drop and the Need for Transfusion in Primary Knee Arthroplasty

**DOI:** 10.7759/cureus.27659

**Published:** 2022-08-03

**Authors:** Fatema H Madan, Ebrahim Khamis, Mohamed Aqeel Alhassan, Maryam Alrashid, Ahmed Saleh, Mohamed Rahma

**Affiliations:** 1 Orthopaedics and Trauma, Salmaniya Medical Complex, Manama, BHR; 2 Orthopaedic Surgery, Salmaniya Medical Complex, Manama, BHR

**Keywords:** osteoarthritis, tranexamic acid, hemoglobin drop, blood transfusion, knee arthroplasty

## Abstract

Background

Blood loss is still a serious adverse effect of total knee replacement (TKR), resulting in Hemoglobin drop and a higher need for blood transfusions. Multiple modalities are used to reduce intra-operative blood loss and hemoglobin drop for this procedure, such as placing a drain intraoperatively and Tranexamic acid (TXA) administration. This study aimed to investigate factors associated with hemoglobin loss and blood transfusion increased demand.

Patients and methods

We retrospectively looked at 223 patients who underwent primary unilateral or staged bilateral knee arthroplasty by a single surgeon from January 2013 until April 2018 in Salmaniya Medical Complex (SMC), Bahrain. We looked into patients' demographics such as age, gender, preoperative hemoglobin and hematocrit, postoperative hemoglobin and hematocrit, drain insertion intra-operatively, and the administration of Intravenous tranexamic acid intra-operatively. Eighty-three patients had a drain inserted intra-operatively, and sixty-nine patients received intra-venous Tranexamic acid during the procedure.

Results

Out of 223 patients, 152 patients were included after applying exclusion criteria. The mean hemoglobin (Hb) loss in patients who had a drain inserted was 2.186 g/dL, while patients who received tranexamic acid had a mean of 1.609 g/dL. Multivariable regression analysis revealed that increased blood transfusion requirements postoperatively were significantly associated with reduced pre-operative Hemoglobin levels (p-value .004). Univariable analysis of the hemoglobin drop showed that the use of TXA would reduce Hb loss while a drain would increase Hb drop (p-value .003 and .002, respectively). Moreover, univariable analysis of blood transfusion requirements showed reduced pre-operative Hb, and placing a drain would increase blood transfusion demand (p-value <.001, .044 respectively), while administration of tranexamic acid would reduce blood transfusion demand (p-value 0.05).

Conclusion

Reducing the need for blood transfusion following primary total knee arthroplasty can be achieved by maintaining preoperative hemoglobin levels, administering tranexamic acid intra-operatively, and avoiding placing an intra-articular drain.

## Introduction

Since the mid-twentieth century, the frequency of knee osteoarthritis has increased, primarily due to the recent increase in life expectancy and high BMI [1]. Subsequently, the number of primary knee replacement procedures is increasing, which has led to more than seven million Americans have undergone hip or knee replacements in 2015. Following these procedures and despite having advanced arthritis, most cases are mobile, which can predict that the current trends will likely continue in the coming decades [2].

Primary knee replacement operations can predispose patients to multiple postoperative risks such as superficial and deep wound infections, wound dehiscence, hematoma formation, deep venous thrombosis, and bleeding requiring transfusion [3]. Various modalities are currently used to reduce intra-operative blood loss for such procedures, such as the use of above-knee tourniquets [4], electrocautery [5], clamping drains [6], adrenaline and saline infiltration [7], and tranexamic acid administration [8].

Tranexamic acid (TXA) is a synthetic competitive fibrinolytic inhibitor of plasmin, plasminogen, and fibrin from combining, and at high concentrations, it directly inhibits plasmin activity [9]. For controlling peri- and postoperative bleeding, TXA has been proven to be safe and efficacious in orthopedic surgery, particularly knee arthroplasty, hip arthroplasty, and spine surgery [10]. TXA can be administered in different methods such as intravenous, topical, and oral [11].

Most bleeding in total knee replacement (TKR) surgeries occurs postoperatively [12]. This bleeding can be associated with the development of wound dehiscence, hematoma, and risk of infection. To aid in reducing the incidence of bleeding and subsequent transfusions, the use of drains kept at the site of surgery intraoperatively during arthroplasty procedures has been used by many surgeons [13].

Although rare, the use of allogenic packed red blood cell (PRBC) transfusions carries multiple risks [14], including the transmission of blood-borne infections where an apparent reduction of the transfusion transmission of hepatitis C virus (HCV) and HIV residual risk to approximately 1:1.2 million and 1:1.5 million, respectively [15] and transfusion-related acute reactions with the rate of 1.6% [16]. 

The aim of the current study is to address factors associated with increased hemoglobin level drop and increased need for Packed Red Blood Cells (PRBCs) blood transfusions.

## Materials and methods

This study was carried out in Salmaniya Medical Complex (SMC), the most prominent public hospital in Bahrain. We have retrospectively looked at the charts of 223 patients who underwent either primary unilateral or staged bilateral knee arthroplasty for osteoarthritis, rheumatoid arthritis, or any other degenerative joint disease from January 2013 to April 2018.

All procedures were performed by one surgeon utilizing the same surgical technique for all patients. In maintaining the hemostasis, the surgeon used to keep an intra-articular closed drainage system for all patients undergoing knee replacement surgery until December 2015. After that, the drain was abandoned and substituted with one gram of intravenous tranexamic acid given preoperatively and 500 mg for intra-articular infiltration just before wound closure.

Patients who underwent simultaneous bilateral arthroplasty, uni-compartmental, or revision knee surgeries were excluded from the study. Patients with coagulopathy and bleeding disorders and patients who had incomplete charts were also excluded from the study. The remaining patients were included in this study, and the following factors were investigated: age, gender, preoperative hemoglobin and hematocrit, postoperative hemoglobin and hematocrit, drain insertion intra-operatively, and the administration of Intravenous tranexamic acid intra-operatively.

All patients were admitted electively one day prior to the planned surgery. Preoperative hemoglobin level was assessed for all patients. The Hb level was routinely checked on the first postoperative day. All patients were typically kept for five to six days before discharge.

A high-thigh pneumatic tourniquet was applied to all patients, using a fixed pressure of 350 mmHg. The tourniquet was deflated after the closure of the wound, and dressings with a compression bandage were applied.

Patients were all ambulated on the first postoperative day. Postoperatively, routine deep vein thrombosis prophylaxis was used for all patients and included intermittent foot compressors and a daily preventive dosage of low molecular weight heparin administered subcutaneously (LMWH). These precautions were maintained until the patient was discharged.

Statistical analysis

Analysis was conducted using SPSS (IBM Corp. Released 2017. IBM SPSS Statistics for Windows, Version 25.0. Armonk, NY: IBM Corp). To summarize the data, descriptive statistics were utilized. Linear regression was performed to determine the significance and correlation of the parameters against Hb drop and the need for transfusion. Moreover, Logistic regression was used to assess the effect of tranexamic acid on the rate of blood transfusion. For both objectives, univariable and multivariable regression analysis was done.

Ethical considerations

All data were collected from patient records through the Salmaniya Medical Complex electronic database (I-SEHA) or physical files for patients who underwent procedures before the use of electronic records. The ethical research committee gave their approval to this project.

## Results

The study included two hundred and twenty-three patients who had a primary cemented total knee replacement between 2013 and 2018 by a single experienced surgeon. Seventy-one patients were excluded due to the following reasons: three patients underwent simultaneous bilateral TKR, two patients received blood transfusion preoperatively due to low Hb, Forty patients did not have their Hb value checked postoperatively, sixteen patients had incomplete charts, five patients did not have either a drain or TXA and five patients had a drain inserted and received TXA.

The remaining 152 patients included 134 women (88.16%) and 18 men (11.84%). The mean age for patients was 62.3 ± 7.8 years at the time of surgery, with patients ranging from 47 to 81 years old (Table 1). Nine patients were known cases of rheumatoid arthritis, while the rest were diagnosed with advanced osteoarthritis. There were 83 patients who had a drain placed intraoperatively and 69 patients who received intravenous Tranexamic acid intra-operatively. Eighteen patients (11.84%) received one or more units of PRBCs transfusion postoperatively, of whom fourteen patients had a drain inserted, and four patients received Tranexamic acid (Table 1).

**Table 1:** Patient demographics, pre-, and post-operative observations HCT: hematocrit, Hb: Hemoglobin, TXA: Tranexamic acid

Patient Specifics	Cohort (n=152)
Age	62.3 ±7.8 (47.0-81.0)
Gender	
Female	134 (88.16%)
Male	18 (11.84%)
Pre-operative HCT mean	37.464 ± 3.732 (26.8-48.0)
Pre-operative HB mean	11.937 ±1.335 (8.1-16.1)
Postoperative HB mean	10.014 ±1.41 (6.9-13.9)
Transfusion /patient	18 (11.84%)
drain	14 (77.8%)
TXA	4 (22.2%)
Hb drop	
drain /mean	2.186 g/dL
TXA	1.609 g/dL

To decrease the impact of confounding variables, multivariable regression analysis was used to analyze the factors affecting hemoglobin drop (Table 2) and blood transfusion requirements (Table 3); only reduced pre-operative Hemoglobin level was found to be significant in the multivariable analysis with the increased need for blood transfusion (p-value =.004) (Table 3). Univariable analysis of the hemoglobin drop (Table 2) showed that the use of TXA would reduce Hb loss while a drain would increase Hb drop (p-value .003 and .002, respectively). Moreover, univariable analysis of blood transfusion requirements (Table 3) showed reduced pre-operative Hb, and placing a drain would increase blood transfusion demand (p-value <.001,0.044 respectively), while administration of Tranexamic acid would reduce blood transfusion demand (p-value 0.05).

**Table 2:** Regression analysis of Hb drop (g/dL) Dependent Variable: HB Loss Drop (95% Confidence Interval), *Statistically significant P < 0.05, HCT: hematocrit, Hb: Hemoglobin, TXA: Tranexamic acid

Variable	Univariable		Multivariable	
	Regression Coefficient	p-value	Regression Coefficient	p-value
Age	-0.007	.589	-0.012	.259
Preoperative HB	0.324	< .001*	0.376	.006*
Gender (Male vs. Female)	0.099	.736	-0.501	.073
TXA (Yes/No)	-0.567	.003*	-0.333	.571
Drain (Yes/No)	0.577	.002*	0.275	.642
Pre-Operative HCT	0.097	< .001*	0.029	.555
Transfusion (Yes/No)	0.698	.017*	1.000	< .001*

**Table 3:** Regression analysis of the need for transfusion Dependent Variable:  Transfusion (Yes/No) (95% % confidence level), Statistically significant at P < 0.05, HCT: hematocrit, Hb: Hemoglobin, TXA: Tranexamic acid

Variable	Univariable		Multivariable	
	Regression Coefficient	p-value	Regression Coefficient	p-value
Age	0.150	.633	0.029	.399
Preoperative HB	-0.814	< .001*	-0.752	.004*
Gender (Male vs. Female)	-19.340	.998	-18.599	.998
TXA (Yes/No)	-1.163	.05 *	-1.157	.063
Drain Placement (Yes / No)	1.193	.044*	1.098	.526

The mean drain output was 218.47 ± 158.99 ml, ranging from 0-700 ml; there was no statistical significance between drain output volume and Hb drop or blood transfusion requirements (p-value =.401).

Four patients presented with superficial wound infections treated with intravenous antibiotics following discharge from the hospital. One patient had a drain inserted, and three received Tranexamic acid. No patients presented with deep venous thrombosis or wound dehiscence.

## Discussion

Patients who undergo total knee arthroplasty (TKA) have a high risk of significant blood loss, subsequently leading to a likelihood of blood transfusion, a longer hospital stay, and postoperative complications. Several studies demonstrated the importance of preoperative hemoglobin as a predictive factor for the need for blood transfusion postoperatively [17]. The aforementioned led to several research focused on investigating securing the hemostasis during knee replacement surgeries. Specifically, this paper was designed to investigate factors that predispose to reduced hemoglobin levels postoperatively and subsequently increase the demand for blood transfusion. Similar to other studies such as Noticewala et al. 2012 and Ryan et al. 2019 [17,18], preoperative hemoglobin level was found as a significant factor associated with the need for transfusion.

Although controversial, the placement of a drain is a common procedure in total knee arthroplasty (TKA). The American Academy of Orthopedic Surgeons (AAOS) stated that there is strong evidence against utilizing a drain in TKA because there is no difference in complications or outcomes between patients with or without a drain [19]. However, there are studies that have suggested that it can impose certain disadvantages related to hemoglobin drop. In terms of drain placement, this study showed a significant increase in hemoglobin drop and blood transfusion requirement with drainage. A systematic review of the efficacy of postoperative drainage in TKA also demonstrated that closed suction drain placement is associated with an increase in hemoglobin drop and an increased rate of blood transfusion following it [20]. Comparing the drainage group and the no-drainage group, Watanabe et al. findings also showed that the mean hemoglobin drop was more significant in the drainage group one day after surgery [21]. Nevertheless, there are other studies that argued otherwise due to the results showing no statistically significant differences in hemoglobin levels nor the rate of blood transfusion even throughout the first six weeks postoperatively [22].

Conversely, a statistically significant reduction in hemoglobin drop and blood transfusion requirements was seen when using TXA intra-operatively. This finding is in line with the strong evidence that supports the use of TXA in lowering postoperative blood loss and minimizing the need for postoperative transfusions after total knee arthroplasty [19,23]. Seol et al. 2016, for example, demonstrated a 24% decrease in the need for transfusion with TXA [24]. The effect of TXA on hemoglobin drop is in concurrence with several other studies, such as Fillingham et al., which concluded that TXA is superior to placebo in terms of decreasing blood loss and risk of transfusion [25].

When comparing both methods, although not interchangeable, the mean hemoglobin drop in patients who had a drain inserted was 1.609 g/dL and 2.186 g/dL in patients who had intravenous TXA intra-operatively, carrying statistical differences between the two groups. In contrast, Anoop Jhurani et al. (2016) found no difference in blood loss volume between the placement of a drain and administering TXA, with the mean Hb drop being 3.24g/dL and 3.54 g/dL respectively [26].

We acknowledge certain limitations in this study; the retrospective study design where the lost files and data might act as confounding factors. The study took place in one institution following a single surgeon's practice, which might mask other factors affecting the outcome. Moreover, the indication of blood transfusion varies with the assigned physicians in the ward, either depending on the value of Hb or clinical manifestations related to low Hb. Additionally, no routine duplex ultrasound appointment was given for the included patients, which might cause missed deep venous thrombosis caused by tranexamic acid or immobilization. Furthermore, using different brands of drains and medical personnel handling the drain might predispose some variable results. To reduce the need for blood transfusion for patients undergoing TKA, it is important to provide further research that can combine different practices and make more solid recommendations in the various forms of hemostasis.

## Conclusions

We found that reducing the need for blood transfusion following primary total knee arthroplasty can be achieved by maintaining preoperative hemoglobin levels, administering Tranexamic acid intra-operatively, and avoiding placing an intra-articular drain. Therefore, we recommend that surgeons maintain an adequate hemoglobin level preoperatively.
